# Pricing and Reimbursement Pathways of New Orphan Drugs in South Korea: A Longitudinal Comparison

**DOI:** 10.3390/healthcare9030296

**Published:** 2021-03-08

**Authors:** Jong Hyuk Lee

**Affiliations:** Department of Pharmaceutical Engineering, College of Life and Health Sciences, Hoseo University, Asan 31499, Korea; jhyuk@hoseo.edu; Tel.: +82-41-540-9814

**Keywords:** essential drugs, rare disease, health insurance, health policies, pharmaceutical economics, budget

## Abstract

This study aimed to analyze four current pathways affecting the listing and post-listing prices of new orphan drugs (ODs) in South Korea. These mechanisms were: (1) essential OD, (2) pharmacoeconomic evaluation (PE) waiver OD, (3) weighted average price OD, and (4) PE OD. We analyzed the ratio of the listing price of 48 new ODs to the average adjusted price (AAP) of seven advanced countries and examined the change in the post-listing price. Descriptive statistics were used to analyze the listing and post-listing price changes. The mean and median ratios of the listing price of total new OD to AAP were calculated to be 69.4% and 65.4%, respectively. Essential OD showed the highest mean (93.8%) and median (80.8%) ratios. The mean cumulative price discount rate of the new OD was 7.2% in the third year and 5.7% in the fifth year. The rarity of diseases impacts the listing price of OD, but the political effects of the benefits of OD on the post-listing price of these drugs could not be verified. Further research should be conducted to develop measures that facilitate the practical sharing of budget risks and increase patient access to new ODs.

## 1. Introduction

Research and development (R&D) on orphan drugs (ODs) for rare diseases has become an active field of research owing to the rapid advancement of biotechnology. Nevertheless, there are still many unmet needs in terms of treatment options for rare diseases [1]. Most rare diseases are genetic in origin, and those who suffer from these diseases occasionally experience symptoms soon after birth or in their childhood. In some cases, these diseases have serious consequences that threaten patients’ lives [2,3,4]. In this regard, the development of new ODs can bring about significantly positive changes in patients’ quality of life. However, the unique characteristics of ODs when compared with non-ODs (e.g., low disease prevalence, disease severity, incomplete understanding of the disease pathology, phenotypic heterogeneity, and limited knowledge of the natural history) are regarded as major challenges for R&D [5,6]. On the other hand, government incentives implemented through legislation, such as tax credits and R&D grants, smaller clinical trials, shorter clinical trial times, and higher rates of regulatory success, serve as R&D drivers [7,8]. Moreover, favorable reimbursement, premium pricing, lower marketing costs, and faster uptake are key drivers for commercialization after marketing authorization [7,8]. In particular, the pricing and reimbursement (P&R) processes of ODs are perceived as the final stage for market access, as well as a crucial issue in terms of patient access to medical treatment [9,10]. That is, when new ODs are not reimbursed, patients are unlikely to use these ODs, instead opting out of appropriate medical treatments to avoid the associated financial burden. The issue of the non-reimbursement of new ODs also affects R&D conducted by pharmaceutical companies. The reimbursement of new ODs at high prices enables these companies to retrieve R&D costs and motivates them to participate in OD development through ongoing investment in R&D on new drugs [8,11]. However, from the payer’s perspective, the budget impact of expensive ODs should be considered. Thus, it can be said that a P&R policy related to OD is influenced by various factors, including the political and social environment [12,13].

In countries that have implemented health technology assessment (HTA), the P&R process is conducted with a focus on proving cost-effectiveness. However, in terms of OD, the lack of established endpoints and small patient numbers are barriers to efficient and effective clinical trials [5,14]. For this reason, different conditions are applied to the P&R processes for ODs compared to those for non-ODs in some countries. As these conditions differ from country to country, such differences need to be considered in advance [15,16,17].

In South Korea, various policies on the P&R process for new drugs have been implemented to increase patient access [18,19]. It has been reported that patient access to new oncology drugs has increased through the implementation of such policies, whereas little research on ODs has been conducted [19]. Many countries determine the price of new drugs by referring to the external reference pricing (ERP). Due to this trend, some pharmaceutical companies delay or even refrain from launching new drugs in countries where the price of new drugs is deemed to be too low [20,21,22,23]. The post-listing price has been found to have an international effect in that the price of a new drug in one country can be cut based on a change in the price of the same drug in other countries, even after listing [21]. Given that such price conditions can affect the price determination of new drugs in other countries, an analysis of listing prices and post-listing prices of new drugs in South Korea can have significant and valuable implications. Thus, this study aimed to analyze the current status of the listing and post-listing price changes of new ODs in the South Korean National Health Insurance scheme.

### 1.1. P&R Pathways for New Orphan Drugs in South Korea 

**Figure 1:**
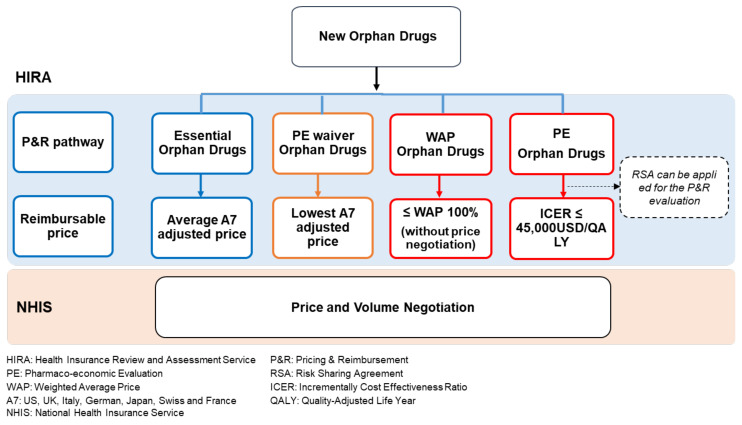
Four pathways for the pricing and reimbursement evaluation of new orphan drugs in the South Korean National Health Insurance Service.

The P&R process for new ODs in South Korea can be classified into four types, each resulting in various additional benefits that are applied to the OD. Particularly, the price of essential ODs that satisfy certain conditions or pharmacoeconomic evaluation (PE) waiver ODs where the PE has been waived is determined based on the price of the same drugs in A7 countries (locally defined reference country group for referring to new drug price: USA, Japan, Germany, France, Switzerland, UK, and Italy) without proving cost-effectiveness. For this reason, these drugs can be listed at high prices through a comparatively simple process [18,19]. As weighted average price (WAP) ODs are listed based on the WAP of alternatives, a higher premium of 10% over the price of non-ODs (90% of WAP) can be applied to them. A risk-sharing agreement (RSA) and flexible incremental cost-effectiveness ratio (ICER) threshold can also be applied to list PE ODs at premium prices through PE. In the PE, the ICER threshold value is generally one gross domestic product (GDP) per capita. However, considering the severity of disease and social influence, the threshold can be increased to 2 GDP per capita (45,000 USD/quality-adjusted life-year (QALY)) for PE ODs [18,19] (Figure 1). 

### 1.2. Price-Cutting System for New Orphan Drugs in South Korea

In South Korea, the National Health Insurance Service (NHIS) operates several systems to reduce the price of drugs listed in the reimbursement drug formulary, which leads to frequent decreases in listing prices. However, the OD prices tend to change less frequently than those of non-ODs because of the benefits applied to the former price even under post-listing price-cutting systems. 

#### 1.2.1. Price–Volume Agreements (PVA)

When the sales volume of a drug increases by 30% or more than the contracted annual volume, the listing price of the drug can decrease by a maximum of 10% through a negotiation between a pharmaceutical company and the NHIS. Under a price–volume agreement (PVA), the benefits for the price of the OD are not applied [24,25].

#### 1.2.2. Price Cutting in the Case of Generic Drug Listings

In the case of non-ODs, when a generic drug is listed, the price of both the original and generic drugs decreases by 53.55% or less. When a generic OD is listed, the price of the original OD is maintained, and the generic OD can be listed at the same price as the original OD [24,25].

#### 1.2.3. Price Cutting with Expanded Indication and Reimbursement Scope

When the indication and reimbursement scope of a listed drug is expanded, its price should be reduced in accordance with the budget impact associated with its wider usage. On the contrary, the price of an OD is not reduced when its scope expands [24,25].

#### 1.2.4. Others

The transaction price of listed drugs is investigated on a biannual basis, and the price of a drug is reduced by a maximum of 10% when its transaction price is lower than the listed price. However, ODs are excluded from such a price reduction. Moreover, the government lowers the price of a listed drug via price re-evaluations that compare its price as determined by other countries, as well as its clinical effectiveness, but ODs are excluded from such drug price re-evaluations [24,25].

## 2. Methods

This study analyzed the status of the listing and post-listing prices of 48 new ODs listed between January 2007 and March 2017 in South Korea. The 48 new ODs were classified according to their respective P&R pathways (i.e., an essential OD, PE waiver OD, PE OD, and WAP OD) based on the results evaluated by the Drug Reimbursement Evaluation Committee (DREC) and those published by the Health Insurance Review and Assessment Service (HIRA) [26].

### 2.1. Analysis of Listing Price of New OD

This study referred to information on the listing price of 48 new ODs in the NHIS drug formulary and analyzed the ratio of the listing price to the average adjusted price (AAP) of A7 countries [27]. The adjusted price applies the ex-factory price to the listing price published on websites that provide information on drug prices in the A7 countries, such as Hokenyaku Jiten (Yakugyo Kenkyukai) in Japan, Vidal in France, Rote Liste in Germany, L’Informatore Farmaceutico in Italy, Arzneimittel Kompendium in Switzerland, the MIMS in the United Kingdom, and the Red Book in the United States. Subsequently, an exchange rate, value-added tax (VAT), and a distribution and transaction range are added to the listing price to calculate and average the adjusted price of the target drugs in each country [28]. The listing prices of the A7 countries were calculated based on the drugs reviewed by the DREC during the previous month [28].

Adjusted price (AP) = (price × ex-factory rate) × exchange rate × (1 + VAT rate) × (1 + distribution margin)

Ex-factory rate: 82.0% for Japan, a separate formula is applied for Germany, and 65% for the other five countries.

VAT rate: 10%.

Distribution margin: 8.7% for high-priced drugs (475 KRW for internal and external application drugs, 4750 KRW or more for injections) and 10.4% for low-priced drugs.

The characteristics of the new ODs (brand and ingredient name, indication, P&R pathway, AAP of the A7 countries, and listing price) are described. Descriptive statistics (mean, standard deviation, median, minimum, and maximum) were used to compare the level of the listing price with the AAP according to the pathway.

### 2.2. Analysis of Post-Listing Price of New ODs

This study examined the change in the post-listing price of 48 new ODs listed between January 2007 and March 2017, for a period from the date of the drug’s listing to 31 July 2019. It also analyzed the cumulative price-cutting rate by year, the time of the first price cut, and its rate. Data published by the HIRA were used to analyze the drug price changes [27]. Moreover, we analyzed the time of the first price cut by classifying ODs into oncology ODs and non-oncology ODs to identify the effects of oncology on the timing of the first price cut. Descriptive statistics (median, minimum, and maximum) were used to analyze the price-cutting rate, the time of the first price cut, and its rate.

## 3. Results

The results of classifying 48 new OD according to P&R pathways indicated that these drugs consisted of 10 essential OD, 11 PE waiver OD, 11 PE OD, and 16 WAP OD. A total of 13 ODs (27.1%) were listed through RSAs. The mean and median ratios of the listing price of a total of 48 new ODs to the AAP were calculated to be 69.4% (SD: ±29.7%) and 65.4% (min: 35.3%, max: 232.4%), respectively. Essential drugs showed the highest mean (93.8%, SD: ±52.5%) and median (80.8%, min: 39.7%, max: 232.4%) ratios. In contrast, WAP OD showed the lowest mean (59.0%, SD: ±21.0%) and median (48.6%, min: 35.3%, max: 93.2%) ratios (Table 1). 

### 3.1. Analysis of Listing Price of New ODs

#### 3.1.1. Essential OD

Among the ten ODs evaluated as essential drugs, two were listed through refund-type RSAs. The listing price of these drugs was 93.8% (mean ± SD: ±52.5%) and 80.8% (median, min: 39.7%, max: 232.4%) of the AAP. In South Korea, new drugs are generally listed at a lower price than the AAP. While two essential drugs were listed at a higher price than AAP, these were the exceptions (Table 2).

#### 3.1.2. Pharmacoeconomic Evaluation (PE) Waiver OD

Among the 11 ODs evaluated as PE waiver OD, seven ODs were listed through RSAs based on the expenditure cap type. The listing prices of these drugs were 65.1% (mean, SD: ±10.0%) and 65.4% (median, min: 46.9%, max: 86.2%) of the AAP. When an OD is evaluated as a PE waiver drug, the drug is exempted from proving cost-effectiveness. Therefore, an expenditure cap type RSA was signed to share the budget impact (Table 3).

#### 3.1.3. Pharmacoeconomic Evaluation (PE) OD

Among the 11 ODs evaluated as PE OD, four ODs were listed through refund-type RSAs. The listing price of these drugs was 65.8% (mean, SD: ±13.5%) and 66.1% (median, min: 40.4%, max: 86.7%) of the AAP (Table 4). 

#### 3.1.4. Weighted Average Price (WAP) OD

The listing price of 16 ODs evaluated as WAP OD was 59.0% (mean, SD: ±21.0%) and 48.6% (median, min: 35.3%, max: 93.2%) of the AAP. If an alternative exists, it is not subject to an RSA in South Korea. Therefore, none of these drugs were listed through RSAs (Table 5).

### 3.2. Analysis of Post-Listing Price of New ODs 

Of the 48 new ODs, 22 were discounted within the price monitoring period (listing date ~ 07.31.2019). The median cumulative discount rate of the 48 new ODs was 7.2% (range: 1.6–40.8) in the third year and 5.7% (range: 0.4–16.6) in the fifth year. In other words, 12 (12/33, 36.4%) and 8 (8/14, 57.1%) ODs showed a median price discount of 7.2% after three years of listing and 5.7% after five years of listing, respectively. In the 10th year, 3 ODs were discounted by a median of 5.3% (range: 5.0–39.3) (Table 6). 

We classified 22 ODs with reduced prices as oncology ODs and non-oncology ODs. We also analyzed the time elapsed until the first price cut after listing and its rate. The result of the analysis regarding the time elapsed before the 22 ODs received their first price cut after listing showed that oncology ODs took 20 months (range: 8.0–83.0) and non-oncology ODs took 25 months (range: 15.0–48.0). The price was discounted faster in the oncology OD group than in the non-oncology OD group. In addition, the median discount rate of the first price cut was 5% for oncology OD (range: 0.4–20.0) and 3.5% for non-oncology OD (range: 0.6–9.1) (Table 7).

## 4. Discussion

As the listing price of new drugs in a country affects prices in other countries through ERP, pharmaceutical companies tend to develop strategies to maintain the global price of these drugs. In particular, an OD that is used to treat a rare disease and lacks alternatives can be listed at a higher price than its price based on value because of the superior bargaining power of pharmaceutical companies or stay in a non-reimbursable state due to failed price negotiations [11,29]. The government reduces the budget impact of newly listed drugs by cutting the price of these drugs or concluding RSAs based on refund and expenditure cap types [30,31,32]. Direct price cutting of new drugs can be regarded as the most effective method to reduce pharmaceutical expenditures from the payer’s perspective. On the other hand, pharmaceutical companies are concerned about how a change in the price of new drugs could impact the global price of drugs. That said, pharmaceutical companies generally have a superior bargaining position in the P&R process before new drugs are listed, given the absence of alternatives. However, the payer may have a superior position when these drugs are listed; the payer can manage patient access to new drugs and the budget impacts of these drugs through the implementation of post-listing policies. 

South Korea is known to strictly manage the listing price of new drugs through the application of positive listing systems in the P&R process [33,34]. In particular, the listing price of new drugs cannot exceed the prices in other countries or the AAP. After these drugs are listed, their prices can be reduced through various mechanisms [24]. Consequently, price-related issues, such as the appropriateness of the listing price and post-listing price cuts, are constantly raised in South Korea. Pharmaceutical companies argue that the listing price of a new drug is too low and that the post-listing price cut for the drug is excessive. At the same time, the payer and insured may argue that the listing price is too high and that the budget impact of the new drug is significant because of the high post-listing price of the drug [18]. In practice, the decision-making process for determining the price of OD, which is generally set to be high, is likely to be disturbed by the aforementioned issues. In this regard, the government should use the RSA system to effectively reduce financial expenditures.

This study compared the price of new ODs listed in South Korea with the AAP of the listing price of the drugs in A7 countries by classifying the drugs according to the P&R pathways. The results of the comparison indicated that ODs evaluated as essential drugs showed the highest listing price ratio and that ODs evaluated as WAP drugs showed the lowest listing price ratio. Based on this result, it can be said that the rarity of the disease affects the price of the new OD and that the price is also influenced by the severity of the social need for the drug, the bargaining power of the pharmaceutical companies, and the political considerations of the payer due to the rarity of diseases. A previous study that examined the correlation between the rarity of diseases and the price of ODs in European countries reported that the rarity of diseases and the price of OD had an inverse correlation [35]. A standard has not been established to determine an appropriate percentage of the listing price of an OD compared to the external reference price, and further analyses are required to inspect specific cases in which the listing price of OD differs significantly in countries with similar income levels.

A previous study found that the cumulative price-cutting rate of new drugs in their fifth year after listing was 5.6% (median, *n* = 79) and that PVA exerted the most significant effect on the post-listing price of new drugs in South Korea [24]. In this study, the cumulative price-cutting rate of new OD products in their fifth year after listing was 5.7% (median, *n* = 8). As a result, the effects of the political benefits applied to post-listing price cuts could not be verified. In other words, the effects of benefits for ODs were not observed because the benefits for ODs were not reflected in the PVA. 

Moreover, this study analyzed the time elapsed until the first price cut after listing and its rate by classifying new ODs into oncology and non-oncology ODs. The analytic result indicated that oncology ODs required less time for the first price cut after listing, which led to a higher price reduction rate because of the effects of the PVA. That is, the budget impact of oncology ODs is more significant than for non-oncology ODs, thereby leading to a swifter price cut based on a higher price reduction rate based on the effects of the PVA. This result also implies that non-oncology ODs need to be revised more flexibly in the P&R process because their budget impact can be predicted more easily and is less significant. In addition, the refund-type RSA, instead of a direct price reduction, could be applied to the PVA to verify the political effects of the benefits of ODs under the post-listing price-cutting systems.

This study empirically analyzed the listing and post-listing price of new ODs recently listed in South Korea. However, the study has limitations in that it used the AAP to analyze the listing price of a new OD. In European countries, the published price of new drugs is unlikely to be the effective price due to the RSA [32]. In terms of drug prices in the United States, a previous study reported that rebates were excluded from the price of drugs listed in the “RED BOOK” and that an accurate net price cannot be identified [36]. In consideration of this issue, it may not be appropriate to compare these directly because not only is the actual net price of A7 countries not precisely known, but also many ODs that were analyzed in this study were listed through refund-type RSAs. 

## 5. Conclusions

A P&R policy has been implemented to apply benefits to the listing and post-listing price of OD in South Korea. The rarity of diseases affects the listing price of the OD. However, the political effects of the benefits of an OD on the post-listing price of these drugs could not be verified. A P&R policy on expensive ODs should consider a number of factors, such as cost-effectiveness, budget impacts, patient access to treatment, and R&D for new ODs. The budget impacts of ODs can be predicted easily and are less significant due to the small number of patients requiring these drugs. Therefore, they need to be revised more flexibly in the P&R process for ODs. Further discussions should also be conducted to develop measures for managing the listing price of ODs through the application of the RSA scheme instead of direct price management, which facilitates the practical sharing of budget risks and increases patient access to innovative new ODs.

## Figures and Tables

**Table 1:** Categorization of new orphan drugs according to pricing and reimbursement pathways in South Korea (listed during the period January 2007–March 2017).

Categorization	Total	Essential Orphan Drug	PE Waiver Orphan Drug	PE Orphan Drug	WAP Orphan Drug
No. of orphan drug	48	10	11	11	16
No. of RSA orphan drug	13	2	7	4	0
KR vs. AAP ratio (%)					
Mean (SD)	69.4 (±29.7)	93.8 (±52.5)	65.1 (±10.0)	65.8 (±13.5)	59.0 (±21.0)
Median (minimum, maximum)	65.4 (35.3, 232.4)	80.8 (39.7, 232.4)	65.4 (46.9, 86.2)	66.1 (40.4, 86.7)	48.6 (35.3, 93.2)

PE: pharmacoeconomic evaluation, WAP: weighted average price, RSA: risk-sharing agreement, KR: listing price in Korea, AAP: average adjusted price of A7 countries (USA, Japan, Germany, France, Switzerland, UK, and Italy), SD: standard deviation.

**Table 2:** New orphan drugs evaluated as essential drugs in South Korea (listed during the period January 2007–March 2017).

No	Brand Name	Indication	Listing Date (Month/Date/Year)	RSA Type	List Price (KRW)	Strength	KR vs. AAP Ratio (%)
	(Generic Name)				List Price (USD)		
1	Cystadane	Homocystinuria	12/01/2007	NA	313,019	180 g	232.4
	(betaine anhydrous)				284.6		
2	Sprycel	Chronic myeloid leukemia	06/01/2008	NA	55,000	70 mg	89.3
	(dasatinib)				50.0		
3	Naglazyme	Mucopolysaccharidosis VI	01/01/2009	Refund	1,614,000	5 mg	79.2
	(galsulfase)				1467.3		
4	Elaprase	Mucopolysaccharidosis II	01/01/2009	NA	2,790,000	6 mg	98.8
	(idursulfase)				2536.4		
5	Myozyme	Pompe disease (GAA deficiency)	05/01/2009	NA	705,000	50 mg	82.5
	(alglucosidase alfa)				640.9		
6	Zavesca	Type 1 Gaucher disease	12/01/2009	NA	98,766	100 mg	65.2
	(miglustat)				89.8		
7	Inovelon	Lennox–Gastaut syndrome	05/01/2010	NA	1280	400 mg	39.7
	(rufinamide)				1.2		
8	Remodulin	Pulmonary arterial hypertension	12/01/2010	NA	5,720,000	2.5 mg/mL	62.5
	(treprostinil sodium)				5200.0		
9	Soliris	Paroxysmal nocturnal hemoglobinuria	10/01/2012	Refund	7,360,629	300 mg	110.0
	(eculizumab)				6691.5		
10	Carbaglu	NAGS (N-acetylglutamate synthase) deficiency	01/01/2015	NA	98,000	200 mg	78.1
	(carglumic acid)				89.1		

Exchange rate: 1 USD = 1100 KRW (Korea Won). NA: not applicable; RSA: risk-sharing agreement; KR: list price in Korea; AAP: average adjusted price of A7 countries (USA, Japan, Germany, France, Switzerland, the UK, and Italy).

**Table 3:** New orphan drugs evaluated as pharmacoeconomic evaluation (PE) waiver pathway in South Korea (listed during the period January 2007–March 2017).

No.	Brand Name	Indication	Listing Date (Month/Date/Year)	RSA Type	List Price (KRW)	Strength	KR vs. AAP Ratio (%)
	(Generic Name)				List Price (USD)		
1	Caprelsa	Medullary thyroid cancer	11/01/2015	Expenditure cap	139,800	300 mg	55.1
	(vandetanib)				127.1		
2	Adcetris	Hodgkin lymphoma	02/01/2016	NA	3,262,400	50 mg	69.7
	(brentuximab vedotin)				2965.8		
3	Vimizim	Morquio A syndrome	06/01/2016	Expenditure cap	1,019,100	5 mg	86.2
	(elosulfase alfa)				926.5		
4	Imbruvica	Chronic lymphocytic leukemia	06/01/2016	NA	65,257	140 mg	69.3
	(ibrutinib)				59.3		
5	Zykadia	Non-small-cell lung cancer	08/01/2016	NA	40,805	150 mg	60.8
	(ceritinib)				37.1		
6	Blincyto	Acute lymphoblastic leukemia	10/01/2016	NA	2,480,000	35 ug	71.0
	(blinatumomab)				2254.5		
7	Diterin	Hyperphenylalaninemia	01/01/2017	Expenditure cap	20,421	100 mg	65.4
	(sapropterin dihydrochloride)				18.6		
8	Defitelio	Veno-occlusive disease	06/01/2017	Expenditure cap	380,300	200 mg	46.9
	(defibrotide)				345.7		
9	Zelboraf	Melanoma	07/01/2017	Expenditure cap	27,200	240 mg	62.7
	(vemurafenib)				24.7		
10	Lynparza	Advanced ovarian cancer	10/01/2017	Expenditure cap	10,510	50 mg	61.0
	(olaparib)				9.6		
11	Meqsel	Non-small-cell lung cancer	11/01/2017	Expenditure cap	166,681	2 mg	68.6
	(trametinib)				151.5		

Exchange rate: 1 USD = 1100 KRW (Korea Won). NA: not applicable; RSA: risk-sharing agreement; KR: list price in Korea; AAP: average adjusted price of A7 countries (USA, Japan, Germany, France, Switzerland, the UK, and Italy).

**Table 4:** New orphan drugs evaluated as pharmacoeconomic evaluation (PE) pathway in South Korea (listed during the period January 2007–March 2017).

No.	Brand Name	Indication	Listing Date (Month/Date/Year)	RSA Type	List Price (KRW)	Strength	KR vs. AAP Ratio (%)
	(Generic Name)				List Price (USD)		
1	Torisel	Renal cell carcinoma	01/06/2011	NA	793,000	30 mg	60.9
	(temsirolimus)				720.9		
2	Trisenox	Acute promyelocytic leukemia	01/06/2011	NA	373,000	10 mg/mL	64.3
	(arsenic trioxide)				339.1		
3	Xtandi	Castration-resistant prostate cancer	01/11/2014	Refund	29,000	40 mg	66.1
	(enzalutamide)				26.4		
4	Jakavi	Myelofibrosis	01/03/2015	NA	56,100	20 mg	57.0
	(ruxolitinib)				51.0		
5	Xalkori	Non-small-cell lung cancer	01/05/2015	Refund	124,000	250 mg	86.7
	(crizotinib).				112.7		
6	Sirturo	Multidrug-resistant tuberculosis	01/05/2015	NA	158,000	100 mg	82.1
	(bedaquiline fumarate)				143.6		
7	Tysabri	Multiple sclerosis	01/10/2015	NA	1,370,000	300 mg	51.4
	(natalizumab)				1245.5		
8	Pirespa	Idiopathic pulmonary fibrosis	30/10/2015	Refund	5750	200 mg	40.4
	(pirfenidone)				5.2		
9	Lemtrada	Multiple sclerosis	01/11/2015	NA	10,371,700	12 mg	78.5
	(alemtuzumab)				9428.8		
10	Gazyva	Chronic lymphocytic leukemia	01/04/2017	NA	4,177,600	1000 mg	69.5
	(obinutuzumab)				3797.8		
11	Pomalyst	Multiple myeloma	09/06/2017	Refund	394,300	4 mg	66.8
	(pomalidomide)				358.5		

Exchange rate: 1 USD = 1100 KRW (Korea Won). NA: not applicable; RSA: risk-sharing agreement; KR: list price in Korea; AAP: average adjusted price of A7 countries (USA, Japan, Germany, France, Switzerland, the UK, and Italy).

**Table 5:** New orphan drugs evaluated as weighted average price (WAP) pathway in South Korea (listed during the period January 2007–March 2017).

No.	Brand Name	Indication	Listed Date (Month/Date/Year)	RSA Type	List Price (KRW)	Strength	KR vs. AAP Ratio (%)
	(Generic Name)				List Price (USD)		
1	Dacogen	Myelodysplastic syndrome	08/01/2008	NA	772,220	50 mg	72.1
	(decitabine)				702.0		
2	Volibris	Pulmonary arterial hypertension	11/01/2011	NA	53,500	10 mg	39.3
	(ambrisentan)				48.6		
3	Tasigna	Chronic myelogenous leukemia	12/01/2011	NA	23,050	200 mg	41.1
	(nilotinib)				21.0		
4	Stribild	Hiv	02/01/2014	NA	27,750	150 mg	44.2
	(elvitegravir/cobicistat/emtricitabine/tenofovir)				25.2		
5	Aubagio	Multiple sclerosis	08/01/2014	NA	38,200	14 mg	42.5
	(teriflunomide)				34.7		
6	Replagal	Fabry disease	08/01/2015	NA	2,403,718	3.5 mg	93.2
	(agalsidase alfa)				2185.2		
7	Vpriv	Type-1 Gaucher disease	08/01/2015	NA	1,888,000	400 unit	86.2
	(velaglucerase alfa)				1716.4		
8	Deltyba	Multidrug-resistant tuberculosis	11/01/2015	NA	39,800	50 mg	91.2
	(delamanid)				36.2		
9	Revolade	Immune thrombocytopenia	03/01/2016	NA	35,443	50 mg	63.1
	(eltrombopag)				32.2		
10	Nplate	Immune thrombocytopenia	03/01/2016	NA	735,000	500 ug	35.3
	(romiplostim)				668.2		
11	Tecfidera	Multiple sclerosis	07/01/2016	NA	20,558	240 mg	44.5
	(dimethyl fumarate)				18.7		
12	Fytarex	Multiple sclerosis	06/01/2017	NA	40,176	0.5 mg	41.6
	(fingolimod)				36.5		
13	Kynteles	Ulcerative colitis, Crohn’s disease	08/01/2017	NA	1,492,000	300 mg	46.2
	(vedolizumab)				1356.4		
14	Tafinlar	Melanoma	09/01/2017	NA	41,765	75 mg	61.4
	(dabrafenib)				38.0		
15	Alecensa	Non-small-cell lung cancer	10/01/2017	NA	20,453	150 mg	51.0
	(alectinib)				18.6		
16	Cerdelga	Gaucher disease type 1	11/01/2017	NA	469,000	84 mg	90.7
	(eliglustat)				426.4		

Exchange rate 1 USD = 1100 KRW (Korea Won). NA: not applicable; RSA: risk-sharing agreement; KR: list price in Korea; AAP: average adjusted price of A7 countries (USA, Japan, Germany, France, Switzerland, the UK, and Italy).

**Table 6:** Cumulative price-cutting rate of new orphan drugs after listing by year.

No. of Years *	No. of New ODs **	No. of Price Cuts	Ratio (%)	Median Cumulative Price-Cutting Rate (%) (Min-Max)
1	48	2	4.3	6.4 (1.9–10.9)
2	42	12	28.6	5.0 (1.5–10.9)
3	33	12	36.4	7.2 (1.6–40.8)
4	21	10	47.6	6.2 (1.1–16.6)
5	14	8	57.1	5.7 (0.4–16.6)
6	12	7	58.3	5.0 (0.4–18.1)
7	11	7	63.6	5.0 (0.5–9.3)
8	8	4	50.0	6.5 (5.0–12.7)
9	5	3	60.0	5.1 (5.0–12.7)
10	3	3	100.0	5.3 (5.0–39.3)

* Based on the month of listing, one year was calculated as a period of 12 months after the listing. ** In the case that a product was discounted in the first year but did not get discounted in the second and third year, the product will still be included in the number of products in the second and third year.

**Table 7:** Time elapsed until the first price cut after listing and its rate of new orphan drugs.

Orphan Drugs					
Oncology			Non-Oncology		
No. of Product	Median Time to First Cut	Median Price Cut Rate	No. of Product	Median Time to First Cut	Median Price Cut Rate
	Month	%		Month	%
11	20.0	5.0	11	25.0	3.5
(8.2%)	(8.0–83.0)	(0.4–20.0)	(8.2%)	(15.0–48.0)	(0.6–9.1)
